# Prediction of survival in amyotrophic lateral sclerosis: a nationwide, Danish cohort study

**DOI:** 10.1186/s12883-021-02187-8

**Published:** 2021-04-17

**Authors:** Anne-Lene Kjældgaard, Katrine Pilely, Karsten Skovgaard Olsen, Anders Hedegaard Jessen, Anne Øberg Lauritsen, Stephen Wørlich Pedersen, Kirsten Svenstrup, Merete Karlsborg, Helle Thagesen, Morten Blaabjerg, Ásta Theódórsdóttir, Elisabeth Gundtoft Elmo, Anette Torvin Møller, Lone Bonefeld, Mia Berg, Peter Garred, Kirsten Møller

**Affiliations:** 1 Laboratory of Molecular Medicine, Department of Clinical Immunology Section 7631, Diagnostic Centre, Rigshospitalet, Ole Maaloesvej 26, DK-2200 Copenhagen N, Denmark; 2 Department of Neuroanaesthesiology, Neuroscience Centre, Rigshospitalet, Copenhagen, Denmark; 3 Institute of Actuarial Science, University of Copenhagen, Copenhagen, Denmark; 4 Department of Neurology, Neuroscience Centre, Rigshospitalet, Copenhagen, Denmark; 5 Department of Neurology, Bispebjerg Hospital, University of Copenhagen, Copenhagen, Denmark; 6 Department of Neurology, Roskilde University Hospital, Zealand University Hospital, Roskilde, Denmark; 7 Department of Neurology, Odense University Hospital, Odense, Denmark; 8 Department of Neurology, Aarhus University Hospital, Aarhus, Denmark; 9 Department of Clinical Medicine, Faculty of Health and Medical Sciences, University of Copenhagen, Copenhagen, Denmark

**Keywords:** ALSFRS-R slope, Amyotrophic lateral sclerosis, Median survival time, Prognostic biomarker

## Abstract

**Introduction:**

Amyotrophic lateral sclerosis (ALS) is a progressive motor neuron disease with great heterogeneity. Biological prognostic markers are needed for the patients to plan future supportive treatment, palliative treatment, and end-of-life decisions. In addition, prognostic markers are greatly needed for the randomization in clinical trials.

**Objective:**

This study aimed to test the ALS Functional Rating Scale-Revised (ALSFRS-R) progression rate (ΔFS) as a prognostic marker of survival in a Danish ALS cohort.

**Methods:**

The ALSFRS-R score at test date in association with duration of symptoms, from the onset of symptoms until test date, (defined as ΔFS’) was calculated for 90 Danish patients diagnosed with either probable or definite sporadic ALS. Median survival time was then estimated from the onset of symptoms until primary endpoint (either death or tracheostomy). ΔFS’ was subjected to survival analysis using Cox proportional hazards modelling, log-rank test, and Kaplan-Meier survival analysis.

**Results and conclusions:**

Both ΔFS’ and age was found to be strong predictors of survival of the Danish ALS cohort. Both variables are easily obtained at the time of diagnosis and could be used by clinicians and ALS patients to plan future supportive and palliative treatment. Furthermore, ΔFS’, is a simple, prognostic marker that predicts survival in the early phase of disease as well as at later stages of the disease.

## Introduction

Amyotrophic lateral sclerosis (ALS) is a rare motor neuron disease of unknown origin, which causes progressive destruction of the motor neurons leading to loss of skeletal muscle function and eventually impaired speaking, swallowing, walking, and breathing. It is a devastating neurodegenerative disease associated with frontotemporal dementia in up to 15% of the patients [1]. Usually, it follows a rapidly fatal course with a median survival time of 24–48 months, albeit with wide variation [2].

Once the diagnosis is established, prognostication is essential to help patients in deciding what they wish to accomplish with their remaining life time, as well as choosing levels of treatment and care. Risk factors for an aggressive progression of the disease for the newly diagnosed ALS patient include body mass index (BMI), symptom onset site, forced vital capacity (FVC%), age, sex, levels of neurofilaments (NFs) in blood and cerebrospinal fluid (CSF), the presence of frontotemporal dementia, and the level of daily functions at the time of diagnosis [3, 4].

By many clinicians, the level of daily functions is estimated by the ALS Functional Rating Scale-Revised (ALSFRS-R) [5]. The ALSFRS-R score is calculated after an interview with the patient or their caregivers. The ALSFRS-R score rates 12 daily activities from 0 through 4, where 0 equals no function at all, and 4 equals normal function. The total score thus ranges from 0 through 48; the decline in ALSFRS-R during the course of the disease has been shown to be curvilinear [6]. This score may aid, if applied in the early state of the disease, in predicting the length of survival [7].

Although ALSFRS-R is scored routinely by ALS clinicians as well as in clinical trials, some authors have suggested that calculating the rate of symptom progression may be a better predictor of survival. The ALSFRS-R progression slope (ΔFS), which normalizes the ALSFRS-R by the duration of symptoms, has been suggested as a predictor of survival [8]. However, others have suggested that the ALSFRS-R progression slope might not be applicable to all ALS populations due to cultural differences [9]. Thus this study aimed to assess the predictive value of ΔFS in a Danish ALS cohort.

## Material and methods

We included 90 patients who had been diagnosed with either probable or definite sporadic ALS according to the Awaji criteria [10] and the El Escorial Revised criteria [11] at one of five participating outpatient ALS clinics in Denmark. The patients were enrolled from 23rd of February, 2016 to 23rd of May, 2018. One hundred nine patients were screened for enrolment. Of those, 19 patients were excluded as they did not meet the criteria of either probable or definite sporadic ALS; this included two patients with familial ALS as diagnosed by genetic workup and the family history. Follow-up data were censored on the 16th of June, 2020. The following demographic information was collected for the present study: age at onset of disease, sex, time from onset of symptoms to diagnosis, symptom onset site, history and symptoms of cognitive impairment as recorded by the neurologist who was responsible for the patient workup, riluzole treatment, and ALSFRS-R score at the data collection day. The ALSFRS-R score was based on an interview with the ALS patients with or without close relatives present. This interview was conducted by the primary investigator. Patients were followed until death or onset of invasive mechanical ventilation, whichever occurred first (the primary endpoint).

### Calculations and statistical analyses

ΔFS is calculated as [8]:

$$ \varDelta FS=\frac{48-\left(\mathrm{Total}\ \mathrm{ALSFRS}-\mathrm{R}\ \mathrm{score}\ \mathrm{at}\ \mathrm{initial}\ \mathrm{assessment}\right)}{\mathrm{Time}\ \mathrm{from}\ \mathrm{onset}\ \mathrm{of}\ \mathrm{symptoms}\ \mathrm{to}\ \mathrm{initial}\ \mathrm{assessment}\ \left(\mathrm{months}\right)} $$

However, because some patients were included in the present study after the time of diagnosis, and we did not have access to the original ALSFRS-R score, we used a modified variable (ΔFS’) calculated at the time of inclusion into the study as:

$$ \Delta  F{S}^{\prime }=\frac{48-\left(\mathrm{Total}\ \mathrm{ALSFRS}-\mathrm{R}\ \mathrm{score}\ \mathrm{at}\ \mathrm{assessment}\ \mathrm{at}\ \mathrm{test}\ \mathrm{date}\right)}{\mathrm{Time}\ \mathrm{from}\ \mathrm{onset}\ \mathrm{of}\ \mathrm{symptoms}\ \mathrm{to}\ \mathrm{assessment}\ \mathrm{at}\ \mathrm{test}\ \mathrm{date}\left(\mathrm{months}\right)} $$

For all survival analyses described hereafter, the primary endpoint was the time of death or of initiation of invasive mechanical ventilation, whichever came first. All covariates represented by continuous data were dichotomized around the median to a “high” or a “low” value. The significance level was set at < 0.05. First, we conducted a survival analysis in a univariate Cox’ proportional hazards ratio model, analyzing the effect of age at onset of disease, sex, time from onset of symptoms to diagnosis, onset site, ALSFRS-R score on the inclusion date, and ΔFS’, respectively. Next, the covariates that were found in the univariate analysis to be associated with survival time were analyzed in a multivariate regression analysis (multiple Cox’ proportional hazards model). The covariates that were independently associated with survival time according to this analysis underwent Kaplan-Meier survival analyses with the cut-off at the median value. Log-rank test and Cox’ proportional hazards model, were applied to disclose differences between the groups of each covariate. To evaluate the demography of the remaining covariates, the two groups, analyzed in the Kaplan-Meier plot, were subjected to χ^2^- test or t-test to disclose any imbalanced distributions. For ΔFS’, an additional Kaplan-Meier survival analysis was conducted dividing the group into three subgroups according to two arbitrary cut-off values as described by Kimura and colleagues [8].

## Results

### Demography of a Danish ALS cohort

Upon exclusion of patients who ultimately were diagnosed with a disease other than ALS, 90 patients (39 females, 51 males) with either probable or definite sporadic ALS were included for the survival analyses. Fifty-seven with spinal, 26 with bulbar, 1 with truncal, and 6 with mixed spinal and bulbar onsets of symptoms. An overview of the demographics of the included ALS patients is shown in Table 1. The median survival time from onset of symptoms until the primary endpoint (death or initiation of invasive mechanical ventilation) was 36 months. The median survival time from time of diagnosis until the primary endpoint was 14 months. At censor date (15th of June, 2020), 69 patients had reached the primary endpoint (58 died without receiving invasive mechanical ventilation, 11 patients received invasive mechanical ventilation via tracheostomy). Among the group of 58 that died without receiving invasive mechanical respiratory support, 30 received non-invasive ventilation.

**Table 1:** Demographic information about the Danish ALS cohort

	All	Group 1 (slow progression)	Group 2 (fast progression)	***p***-value
ΔFS’ (mean ± SD)	0.9 (±0.9)	0.4 (±0.2)	1.4 (±1.0)	1.4e-8
No. of patients	90	45	45	–
Age at onset, yrs. (mean ± SD)	62.9 (±11.5)	62.3 (±12.6)	63.4 (±10.3)	0.64
Sex, F/M	39/51	23/22	16/29	0.62
Bulbar onset	26	16	10	0.40
Spinal onset	57	26	31	0.64
Truncal onset	1	1	0	–
Mixed spinal and bulbar onsets	6	3	3	–
Onset to diagnosis, mo (mean ± SD)	16.6 (±11.5)	22.3 (±12.8)	10.8 (±5.8)	9.0e-5
Onset to test date, mo (mean ± SD)	22.4 (±20.7)	31.2 (±24.8)	13.7 (±9.5)	4.9e-5
ALSFRS-R at test date (mean ± SD)	34.5 (±9.3)	36.5 (±9.1)	32.6 (±9.3)	0.043

*ALSFRS-R* ALS functional rating scale – revised, *F/M* Female/male, *mo* Months, *SD* Standard deviation, *yrs.* years

### Median survival time in Danish ALS patients and correlated covariates (Table 2)

The covariates ΔFS’, age at onset of symptom, and time from onset of symptoms to diagnosis were associated with survival time and analyzed further by stepwise, multivariate regression. The results are listed in Table 2. The analyses suggest that time from the onset of symptoms until diagnosis, ΔFS’, and age at the onset of symptoms are associated with survival time from onset of symptoms.

**Table 2:** (A) The results of the univariate survival analysis screening for associations between the median survival time and the covariates available at the test date. (B) The statistically significant covariates from (A) analyzed by stepwise, multivariate regression (Cox proportional hazards model)

(A)			(B)				
Variable	HR	***p***	Variable	HR	***p***	HR coefficient	***p***
Age at onset (yrs)			Age at onset (yrs)				
< 65.2	1	–	< 65.2	1	–	–	–
≥65.2	2.18 (1.34–3.54)	0.0017	≥65.2	2.56 (1.56–4.22)	2.2e-4	1.07 (1.04–1.10)	3.0e-6
**Onset to diagnosis** (mo)			**Onset to diagnosis** (mo)				
< 12.7	1	–	< 12.7	1	–	–	–
≥12.7	0.45 (0.28–0.73)	0.013	≥12.7	0.52 (0.31–0.86)	0.01	0.52 (0.37–0.73)	1.4e-4
**ΔFS’**			**ΔFS’**				
< 0.68	1	–	< 0.68	1	–	–	–
≥0.68	3.66 (2.17–6.18)	1.1e-6	≥0.68	3.3 (1.91–5.69)	1.8e-5	2.19 (1.67–2.88)	1.6e-8
**ALSFRS-R at test date**							
< 36.5	1	–					
≥36.5	0.97 (0.61–1.57)	0.91					
**Sex** (n)							
Female (39)	1	–					
Male (51)	0.92 (0.57–1.48)	0.74					
**Onset site** (n)							
Mixed (6)	1	–					
Truncal (1)	0.95 (0.11–8.2)	0.96					
Bulbar (26)	0.85 (0.32–2.28)	0.75					
Spinal (57	0.78 (0.31–1.98)	0.60					

*ALSFRS-R* ALS functional rating scale, *ΔFS’* ALSFRS-R progression rate, *HR* Hazard ratio, *mo* Months, *p P*-value, *yrs.* years

### The covariates ΔFS’ and age are prognostic predictors for the survival (Fig. 1 and Table 3)

After dividing the ALS cohort into two groups by the 50% quantile of ΔFS’, the median survival time for the group with the slower progression rate (ΔFS’ < 0.68) was 46.5 months as compared with the group with the faster progression rate (ΔFS’ ≥ 0.68) (25.2 months).

**Fig. 1:**
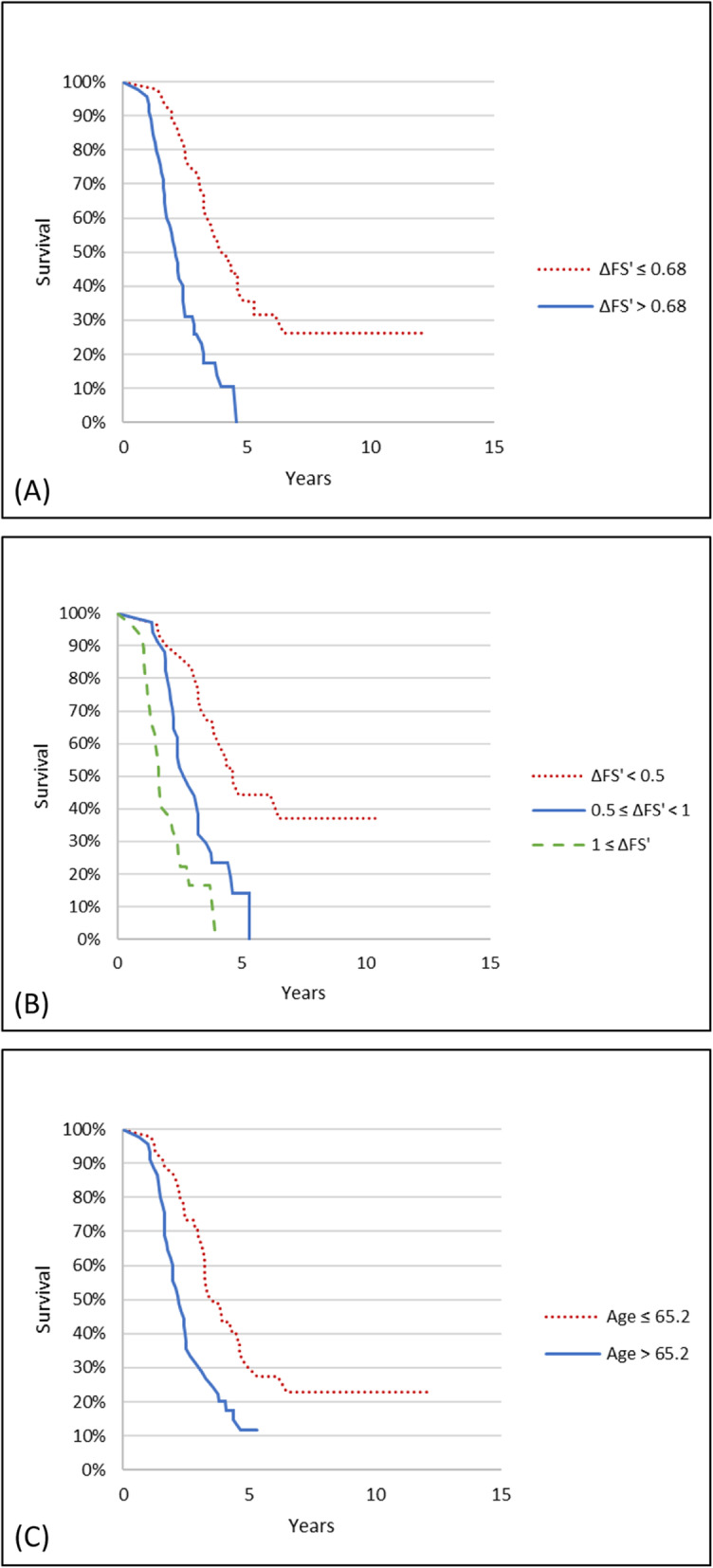
Kaplan-Meier survival plots of the ALS cohort (*n* = 90). **a** The survival plot in accordance with progression rate (ΔFS’) from the onset of symptoms until the primary end point divided by the median ΔFS’ value. **b** The survival plot in accordance with progression rate from onset of symptoms until primary end point divided by two arbitrary cut-off values. **c** The survival plot from onset of symptoms until primary end point in accordance with age at onset of symptoms

**Table 3:** The median survival time from onset of symptoms until the primary end point is calculated in association with ΔFS’ divided into two groups by the 50% quantile, ΔFS’ divided arbitrarily into three groups, and with age divided by the 50% quantile

	Kaplan-Meier survival analysis			Cox proportional hazards		
	No. of pts.	MST	*p* (log-rank)	Hazard Ratio	CI	*p*
**ΔFS’**						
Group 1, < 0.68 (Slow)	45	46.5	–	1	–	–
Group 2, ≥0.68 (Fast)	45	25.2	1.3e-7	3.66	2.17–6.18	1.1e-6
**ΔFS’**						
< 0.5 (Slow)	29	55.5	–	1	–	–
0.5–1.0 (Medium)	34	31.7	5.5e-5	3.12	1.66–5.85	3.9e-4
> 1.0 (Fast)	27	19.8	0.0032	7.50	3.71–15.17	2.1e-8
**Age (yrs)**						
< 65.2	45	40.4	–	1	–	–
≥ 65.2	45	25.9	8.0e-4	2.18	1.34–3.54	0.0017

*CI* Confidence interval, *ΔFS’* ALSFRS-R progression rate, *MST* Median survival time (from onset of symptoms until primary endpoint in months), *p: p*-value, *pts.* Patients, *yrs.* Years

Dividing the ALS cohort by age, the median survival time was 40.2 months for younger (age < 65.2 years) as compared with 25.9 months for older patients (age ≥ 65.2 years).

## Discussion

The ALSFRS-R score is widely acknowledged as a useful marker of the ALS patient’s loss of function concerning activities of daily living. However, it is equally widely acknowledged as a poor predictor of survival [8, 12]. The symptom progression rate in ALS, ΔFS, has been suggested to predict survival, but conflicting results have been reported [8, 9, 12, 13]. In this nationwide study of Danish ALS patients, ΔFS’ as a surrogate for ΔFS emerged as a strong predictor of survival as did age. This indicates that the symptom progression rate may be calculated later than the time of diagnosis without losing predictive power.

The option of mechanical ventilation is variably implemented between countries. We chose a primary endpoint that combined death and tracheostomy (with the aim to initiate invasive mechanical ventilation), which is likely to control for this fact. In contrast, we suggest that the date of initiation of non-invasive ventilation should not be included as a primary endpoint, since the indication for and use of non-invasive treatment varies significantly between centres and patients.

ΔFS has been shown to be a simple, valuable tool to enable balanced randomization of ALS patients in phase II and III clinical trials with regard to the progression rate from onset of symptoms [13]. The use of the change in the ALSFRS-R score during clinical trials to evaluate the effect of the treatment was recently criticized by van Eijk and colleagues, who suggested that a more granular approach to assessing functional deterioration (or absence thereof) in each of the subdomains might be more appropriate; this was intended primarily for future studies of pharmacological treatment [14]. Nonetheless, so far most cohort studies have applied the total score for assessing prognosis, and the approach suggested by van Eijk et al. awaits further validation.

Table 4 shows information from four previous studies that have investigated ΔFS as a predictive marker of survival time. Three of these studies reported that ΔFS was a good or excellent predictor of survival [8, 12, 13]. The fourth study compared two patient cohorts, one from Ireland and one from Italy found that ΔFS performed well in the Irish cohort [9]. However, it was not an independent predictor of survival in the Italian cohort. The authors suggested cultural differences as an explanation for their finding. As an example, in study 4, crude survival rates were used; thus, differences in the use of mechanical ventilation may have differed between cohorts. Furthermore, the inclusion criteria differed between studies. Thus, the four studies included patients with different degrees of probability of ALS (i.e., suspected, possible, probable, and definite ALS); because patients with other types of motor neuron disease have different prognoses [15], their inclusion into the cohorts may have affected survival.

**Table 4:** An overview of previous studies of ΔFS

Reference	No. of ALS Patients (pts)	Primary end point	Methods	Conclusions
Kimura et al. (2006) [8]	82 sALS (15 possible, 32 probable, and 35 definite)	Death or tracheostomy or non-invasive ventilation	Mean ΔFS based on ALSFRS-R score on time of diagnosis. Two groups: ΔFS < 0.67 vs ΔFS ≥ 0.67 Three groups (arbitrary cut-off): ΔFS < 0.5 vs 0.5 ≤ ΔFS < 1 vs ΔFS ≥ 1	Risk of death increased progressively from lowest to highest ΔFS. ΔFS is a good independent predictor of survival time.
Gordon et al. (2006) [13]	112 ALS patients (not further specified)	6-month change in ALSFRS-R.	ΔFS based on ALSFRS-R score on time of diagnosis Two groups: ΔFS < 0.5 vs ΔFS ≥ 0.5	ΔFS is an excellent predictor of the progression rate at time of diagnosis and for stratification in clinical trials.
Elamin et al. (2015) [9]	204 Irish patients 122 Italian patients (possible, probable or definite ALS)	Survival time from initial visit (until death or census date at least 50 months after initial visit)	ΔFS based on ALSFRS-R score on time of inclusion into study Four groups: ΔFS < 0.25 vs 0.25 ≤ ΔFS < 0.49 vs 0.50 ≤ ΔFS < 0.99 vs ΔFS ≥ 1.00	ΔFS for Irish cohort good as independent predictor but a poor predictor for Italian cohort
Labra et al. (2016) [12]	164 patients (suspected, probable, possible or definite)	Survival from initial visit (until death)	Three groups: ΔFS < 0.47 vs 0.47 ≤ ΔFS < 1.11 vs ΔFS ≥ 1.11	ΔFS is a simple, robust independent prognostic biomarker usable for clinical trials.

*ALSFRS-R* ALS functional rating scale revised, *ΔFS* ALSFRS-R progression rate

Our finding that ΔFS’ performed equally well whether the cohort was divided into two or three groups concurs with the original study by Kimura et al.(2006) [8].

As opposed to our findings, Elamin et al. (2015) [9] reported that the age at symptom onset did not predict the length of survival. The effect of age on survival may be partially caused by the fact that age correlates negatively with physical abilities beyond daily living activities. Thus, we suggest that physical strength and hence, the physical reserve is more significant in younger patients. Moreover, end-of-life decisions may be influenced by age; thus, older ALS patients or their caregivers may be more likely to opt out of mechanical ventilation than younger patients.

In addition to these four studies, other groups have also attempted to create predictive models in ALS with progression rate as one of the predictors [16–18]. Of these, the study of Westeneng et al. stands out by its multinational design and cohort size [16]. This study included 11,475 ALS patients from 9 countries and used a multivariable approach to identify eight predictors of progression to tracheostomy, noninvasive ventilation, or death. These predictors included progression rate, with a reported hazard ratio of 3.19 (95% CI, 2.71–3.75). Besides the obvious difference in size and the fact that this study was conducted in several European countries, compared to our study it also included patients with possible ALS, which may have increased the effect size of progression rate.

Recent suggestions of a new set of diagnostic criteria based on both symptoms, clinical and neurophysiological / imaging signs [19], which intend to reduce the diagnostic delay, would likely mean that patients diagnosed with ALS would present a wider spectrum of symptoms (including patients who are currently classified as ‘possible’, ‘probable’, and ‘definite’ ALS), though generally with less functional deterioration over a shorter time course. The usefulness of ΔFS’ in this context remains to be elucidated.

This study has several limitations. First and foremost, this was not a population-based study, as it originated in a study focusing on sampling of biological material. Patients were included from five large centres by personal invitation, and several different types of recruitment bias may have rendered the demography of the participants less representative of the entire cohort of ALS patients in Denmark. Next, in previous studies, ALSFRS-R scoring was done at or close to the time of diagnosis. However, in our study, all ALS patients affiliated with the inclusion sites were invited to participate in this study. Therefore, some of these ALS patients were included at a later stage of the disease compared with previous studies, meaning that the ALSFRS-R score was also obtained later. The ALSFRS-R follows a curvilinear decline over time, although pre- and post-diagnostic slopes are correlated [20]. The inconsistency of ALSFRS-R scoring the patients at different stages of disease could thus be perceived as a limitation of this study. However, our findings indicate that ΔFS’ may be as strong as ΔFS at predicting survival.

Furthermore, our cohort comprised only patients that were diagnosed with probable or definite ALS, compared to previous studies with wider inclusion criteria. Thus the patients in our study would be expected to have a worse diagnosis [21]. On the other hand, as suggested above the present cohort is more likely to represent patients with true ALS.

For the survival analysis, we used cutoffs based on the distribution of ΔFS’ in the present cohort.

It was not within the scope of this study to obtain information about BMI, FVC%, and neuropsychological status. These covariates have also been shown to affect the prognosis of ALS in previous studies. Similarly, important prognostic predictors such as cognitive impairment were recorded by the attending clinicians and were not further defined [3]. Finally, riluzole may be associated with debilitating symptoms such as severe nausea, anorexia, and diarrhoea. Even if riluzole was prescribed by the neurologist in charge, many patients reported low or non-compliance with this treatment. Accordingly, we elected to omit this covariate from the analysis.

## Conclusion

In this Danish ALS cohort study, ΔFS’, age at onset of disease, and time from onset of symptoms until diagnosis emerged as independent predictors of survival. The study substantiates previous findings of ΔFS as a prognostic biomarker, which is easily implemented by neurologists to guide prognosis and decision-making in the initial phase of the disease. Furthermore, the symptom progression rate may predict survival, whether calculated at the time of diagnosis or later during the course of the disease.

## Data Availability

The datasets generated and analysed during the current study are not publicly available due to Danish legislation but are available from the corresponding author (anne-lene.kjaeldgaard@regionh.dk) on reasonable request.

## Abbreviations

ALS: Amyotrophic lateral sclerosis
ALSFRS-R: Amyotrophic lateral sclerosis functional scale revised
BMI: Body mass index
CSF: Cerebrospinal fluid
ΔFS: ALSFRS-R progression rate
EMG: Electromyography
ENG: Electroneuronography
fALS: Familial amyotrophic lateral sclerosis
FVC%: Forced vital capacity
MEP: Motoric evoked potentials
MRI: Magnetic resonance imaging
MST: Median survival time
NF: Neurofilaments
NIV: non-invasive ventilation
PEG: Percutaneous endoscopic gastrostomy
sALS: Sporadic amyotrophic lateral sclerosis
SD: Standard deviation
